# Diversion at the ER: How *Plasmodium falciparum* exports proteins into host erythrocytes

**DOI:** 10.12688/f1000research.1-12.v2

**Published:** 2012-12-07

**Authors:** Karin Römisch

**Affiliations:** 1 Department of Microbiology, Faculty of Biology, Saarland University, Saarbruecken, Germany

## Abstract

Malaria is caused by parasites which live in host erythrocytes and remodel these cells to provide optimally for the parasites’ needs by exporting effector proteins into the host cells. Eight years ago the discovery of a host cell targeting sequence present in both soluble and transmembrane *P. falciparum* exported proteins generated a starting point for investigating the mechanism of parasite protein transport into infected erythrocytes. Since then many confusing facts about this targeting signal have emerged. In this paper, I try to make sense of them.

## The problem

**Figure 1.**
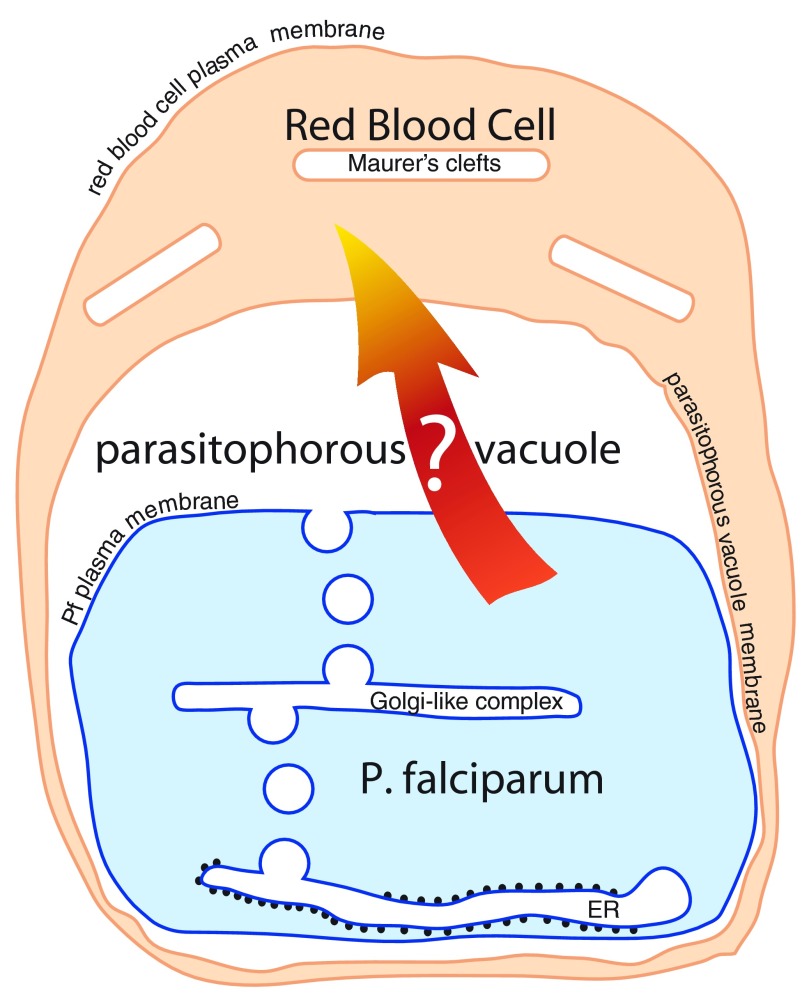
The problem. *P. falciparum* (blue) resides in red blood cells (pale orange) inside a parasitophorous vacuole (white). In order to survive, *P. falciparum* needs to export numerous proteins into the red blood cell, which remodel the host cell to suit the purposes of the parasite. The mechanism by which these proteins are exported is still unclear (Figure modified from Römisch, 2005) ^6^.

*P. falciparum* infects erythrocytes and causes malaria in humans. The parasite resides intracellularly in a parasitophorous vacuole (PV), and exports proteins into the erythrocyte that are important for parasite survival ( Figure 1) ^1, 2^. The identification of a host cell targeting signal in exported *P. falciparum* proteins was an important first step towards understanding the export mechanism, but left cell biologists puzzled: Marti *et al.* (2004) and Hiller and colleagues (2004) identified a short sequence, RxLxE/Q, which is present in many proteins in the *P. falciparum* genome known to be exported from the parasite into the erythrocyte ^3, 4^. This *P. falciparum* protein export element (PEXEL) was found by both groups to be necessary and sufficient for protein export into the host cell ^3, 4^.

What remains unclear to date is the mechanism by which an export signal present in both soluble and transmembrane proteins can mediate transport of both types of protein into the erythrocyte. This issue was debated hotly, but our ideas at the time were limited, because they were solely based on the classical secretory pathway in mammalian cells ^5– 7^.

## The fact(or)s

Since then, a lot more data related to the PEXEL sequence have been generated, but rather than clarify they seem to confuse the issue further. Meanwhile it has been shown that:

- The secretory signal peptides of the exported soluble proteins, which are located 20–40 amino acids upstream of the host cell targeting signals, are not cleaved upon endoplasmic reticulum (ER) targeting ^8, 9^.
- The host cell targeting signal is cleaved and the new N-terminus is N-acetylated at the ER membrane ^9– 11^.
- The protease responsible for the cleavage has been identified ^9– 12^.
- Cleavage by this protease is a prerequisite for transport into the erythrocyte ^9, 10^; if you generate the cleaved N-terminus by modifying the gene and combine it with a cleavable signal peptide, the resulting protein is secreted into the PV and remains there ^9^.
- The uncleaved targeting signal binds PI3P at the ER membrane with the same specificity required for protease cleavage and host cell targeting; the cleaved signal no longer binds PI3P ^13^.
- A putative 'translocator' complex resides in the PV membrane (PVM); it consists of 5 proteins that coprecipitate some of the proteins bearing a host cell targeting signal, but a function of the complex has not been demonstrated in any way, nor has it been investigated whether the association of the complex and the PEXEL proteins is mediated by the PEXEL signal ^14, 15^.

## The hypothesis

**Figure 2.**
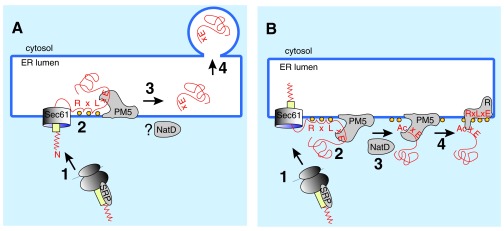
**A**) **The hypothesis.** Most proteins targeted for export into the host cell have a signal sequence (yellow) or transmembrane domain, which leads to their SRP-mediated targeting to the protein translocation channel (Sec61) in the ER membrane of the parasite (1). Many but not all of the *P. falciparum* exported proteins have an N-terminal extension (red zigzag) whose function is unknown. In addition, host cell targeted proteins contain, in a distance of 20–40 amino acids from the signal peptide, a PEXEL sequence (RxLxE), which is also required for binding to phosphoinositol-3-phosphate (PI3P; orange balls) and for cleavage by the ER-membrane associated protease plasmepsin V (PM5). The current hypothesis in the field is that, after signal-peptide mediated translocation into the ER lumen, the PEXEL sequence binds to PI3P in the lumenal face of the ER membrane and is cleaved by PM5 (2). The cleaved protein is released from PM5 (3) and continues through the *P. falciparum* secretory pathway by vesicular transport (4). Note that this model cannot explain the NatD-mediated N-acetylation of the PM5-cleaved N-terminus, because NatD resides in the cytosplasm. **B**) **The alternative**. After SRP-mediated targeting of the protein destined for export to the parasite ER (1) and insertion into the Sec61 channel, the N-terminal extension of the signal peptide (red zigzag) delays signal cleavage, perhaps by preventing reorientation of the signal peptide in the Sec61 channel. This delay in completing translocation allows the RxLxE sequence to bind to PI3P (orange balls) on the cytoplasmic face of the ER membrane, which creates a recognition signal for PM5 and results in proteolytic cleavage (2). Cleavage releases the protein from the translocation machinery and allows N-acetylation by NatD (3). The mature protein is handed over to a PI3P-associated putative transmembrane receptor (R; 4), which may itself be a PEXEL protein.

The authors of the respective papers assume that proteolytic cleavage, N-acetylation, and PI3P binding take place in the ER lumen ( Figure 2A) ^9, 10, 13^. This cannot be right: N-acetylation is a cytosolic modification, based on the biochemical and sequence data characterizing the protease plasmepsin V, its active site is almost certainly on the cytoplasmic face of the membrane, and the only possible location for PI3P at the ER membrane is in the cytosolic leaflet. In detail:

All known enzyme complexes mediating N-acetylation including NatD, which is likely responsible for the N-acetylation of proteolytically cleaved PEXEL proteins, are located in the cytosol ^16^. The presence of a secretory signal sequence indeed strongly reduces the likelihood of proteins being N-acetylated, confirming that N-acetylation does not take place inside compartments of the secretory pathway ^17^.

In the initial characterization of plasmepsin V, Klemba & Goldberg (2005) ^12^ found a hydrophobic region at the N-terminus, which they described as a putative signal sequence. There is no discernible signal peptidase cleavage site C-terminal of this hydrophobic region and indeed Klemba and Goldberg did not observe processing of the N-terminus of plasmepsin V in pulse-chase experiment. Russo and colleagues showed later (2010) ^10^ that fusion of this region of plasmepsin V to a fluorescent reporter protein resulted in ring-shaped staining around the parasite cytoplasm indicative of location in the PV or at the parasite plasma membrane, and suggesting entry of the protein into the ER and transport to the cell surface. Since the N-terminal hydrophobic domain of plasmepsin V is not cleaved ^12^, it is likely a transmembrane signal anchor. Both TMHMM and TopPred predict it to insert into the ER membrane with the N-terminus in the lumen such that the subsequent soluble region containing the active site would be in the cytoplasm. In the same paper Russo and colleagues demonstrated that the C-terminal hydrophobic region of plasmepsin V was required for ER retention of the protein ^10^. Both Russo and colleagues and Klemba and Goldberg describe this region as a transmembrane domain, but Klemba and Goldberg show that 50% of plasmepsin V can be extracted from the membrane by carbonate, pH 11.0 ^12^. A standard feature of a transmembrane protein, however, is that it is resistant to carbonate extraction at pH 11.5 ^18^. Altogether these data suggest that plasmepsin V is tethered to the ER membrane by hydrophobic regions at both termini, and that its active site is in the cytoplasm.

Localization of PI3P has been investigated in yeast and mammalian cells where it is found on early and late endosomes and transiently at the plasma membrane ^19^. PI3P is generated by PI3-kinases on the cytoplasmic leaflet of intracellular membranes and regulates membrane trafficking events ^20^. The localization of the kinase determines the localization of the PI3P patch ^20^. There are no PI3 kinases inside the secretory pathway. The only known example of PI3P occurring at the ER is during formation of autophagosomal membrane precursors, the so-called omegasomes ^21^. Even in this case, PI3P is generated by the Vps34 PI3-kinase in the cytoplasmic leaflet of the ER membrane ^22^.

## The alternative

**Figure 3.**
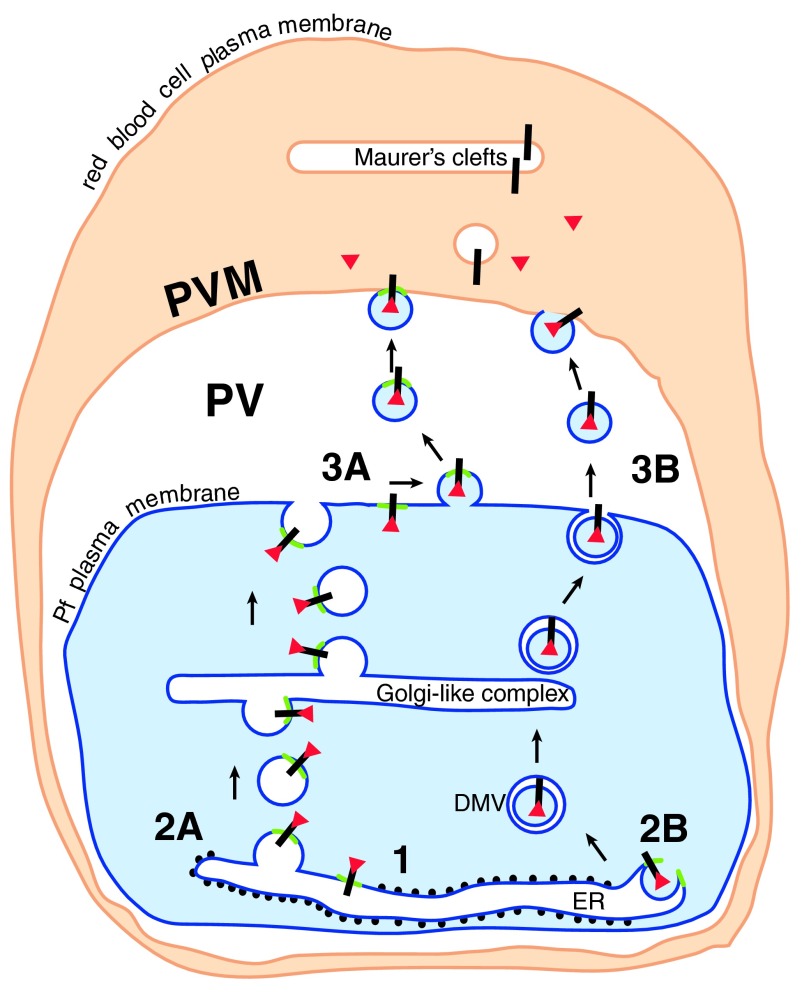
The way out. Cleaved N-acetylated PEXEL proteins (red triangles) associated with their receptor (black bar) could be transported to the cell surface in a complex (1) in one of two ways. Either the receptor/soluble protein complex remains associated with the PI3P patch (green) in the ER membrane. The patch and associated proteins are transported by vesicle budding (2A) and fusion through the parasite secretory pathway to the cell surface, where a further budding event (3A) liberates vesicles containing the PI3P patch and the PEXEL proteins. These vesicles then fuse with the PVM (4A). Alternatively, similar to the formation of omegasomes during autophagy, the PI3P patch may trigger the budding of processed PEXEL protein-containing vesicles into the ER (2B). PEXEL proteins would then be transported through the secretory pathway in double membrane vesicles (DMV), and released into the PV by fusion of the outer membrane with the parasite plasma membrane (3B). The released vesicle may then fuse with the PVM (4B) (Figure modified from Römisch, 2005) ^6^.

An alternative explanation for most of the available data is that their secretory signal peptides target PEXEL proteins to the *P. falciparum* ER, but are inefficiently cleaved - perhaps due to their long N-terminal extensions ( Figure 2B, (1)). This is similar to the biogenesis of some autotransporters in pathogenic *E. coli*, where delayed signal peptide cleavage due to N-terminal signal peptide extension allows the passenger domain to remain unfolded in the periplasm while the porin domain assembles in the *E. coli* outer membrane ^23^. Stuck in the protein translocation channel in the *P. falciparum* ER membrane, the PEXEL protein in transit is oriented such that the host cell targeting signal can bind PI3P at the cytoplasmic face of the ER membrane ( Figure 2B, (2)). Binding creates the cleavage site for the protease plasmepsin V ( Figure 2B, (2)). This possibility has also been mentioned, but not been pursued experimentally, in Bhattacharjee *et al.* ^13^. After cleavage and N-acetylation ( Figure 2B, (3)) the new N-terminus is not released, but the protein remains membrane-tethered or associated with a transmembrane protein ( Figure 2B, (4)) until it reaches the cell surface from where it is transferred to the erythrocyte, perhaps by vesicular transport ( Figure 3).

Unusual biogenesis of a host cell targeted *P. falciparum* membrane protein has already been shown: the protein PfEMP-1 remains peripherally membrane-associated throughout the *P. falciparum* secretory pathway and only becomes transmembrane in the erythrocyte ^24^. But conventional transmembrane proteins, i.e. proteins that become membrane-integrated in the parasite ER membrane, with PEXEL signals also exist ^25^. The biogenesis of a soluble PEXEL protein has not been studied in similar detail to date.

## The key: PI3P in the ER

Recruitment of PEXEL proteins to specific locations within the ER membrane shows interesting parallels to autophagosomal membrane formation at the ER membrane ^21, 22^. As mentioned above, the only known example of PI3P occurring in ER membranes is during the induction of autophagosome formation ^22^. Here the Atg14L protein recruits the Vps34 PI3-kinase to the cytosolic face of ER membranes; during amino acid starvation this leads to the formation of membrane patches or bulges, which seem to be attached to the ER and contain PI3P ^21, 22^. These patches recruit proteins with PI3P-binding domains, promoting formation of so-called omegasomes, which are invaginations into the ER that ultimately lead to pinching off of a crescent-shaped membrane structure ^21, 22^. The mechanism responsible for omegasome formation is not understood ^21, 22, 26^. In order to be recruited to the right place, the proteins binding to PI3P at the cytosolic face of the ER membrane have to contain, in addition to their PI3P-binding domains, a not yet characterized ER-targeting signal ^21^.

So maybe during erythrocyte invasion, which is controlled by IP3 signalling and calcium release from the ER ^27^, a PI3-kinase is recruited to or activated at the *P. falciparum* ER. This PI3-kinase generates PI3P in patches localized in the cytoplasmic leaflet of the ER membrane in proximity to the protein translocation channel (Sec61 channel) or the SRP receptor ( Figure 2B). The signal peptide of soluble PEXEL proteins promotes their targeting to the ER membrane where the signal peptide inserts into the Sec61 channel ( Figure 2B). The as yet unexplained long N-terminal extensions of many of the PEXEL protein signal peptides may lead to their inefficient cleavage by signal peptidase in the ER lumen, similar to delayed signal sequence cleavage in bacterial autotransporters with long N-terminal extensions ^23^. Delayed cleavage may be caused by the extension preventing reorientation to an N-cytoplasmic/C-lumenal topology in the protein translocation channel as shown in Figure 2B ^28^. Alternatively, the N-terminal signal peptide extension may interact with a cytoplasmic domain of the Sec61 channel, which in turn may interfere with signal peptidase access to the cleavage site in the ER lumen (topology shown in Figure 2A). At least some of the N-terminal hydrophobic regions of PEXEL proteins also simply do not contain signal peptidase cleavage sites ^6^. Delayed signal cleavage will lead to prolonged residence of the nascent PEXEL protein in the Sec61 channel, which in turn would allow interaction of the still cytosolic PEXEL signal with PI3P in the cytosolic leaflet of the ER membrane ( Figure 2B). This interaction keeps the protein from entering the ER and thus the conventional secretory pathway. It may also generate a protein conformation recognized by the cytosolically located active site of plasmepsin V and result in cleavage of the PEXEL signal, which liberates the protein from the Sec61 channel, and aborts translocation into the ER ( Figure 2B). Plasmepsin V might itself be a PI3P-binding protein, or interact with the Sec61 channel. After cleavage the cytosolic NatD complex would acetylate the new N-terminus ( Figure 2B). Importantly, the interaction of the PEXEL protein with plasmepsin V leads to a handing over of the cleaved protein to a receptor protein (or complex) in the PI3P-patch ( Figure 2B).

Transmembrane PEXEL proteins are similarly targeted to the Sec61 channel in the ER membrane, but released laterally into the lipid bilayer which allows their cytosolically exposed PEXEL sequence to bind to PI3P and be recruited into the patch ( Figure 2B). In this case the topology of the PEXEL/PI3P interaction may prevent cleavage by plasmepsin V. That transmembrane proteins can be recruited to PI3P in the ER membrane has also been shown during autophagosomal membrane formation at the ER ^21^. One or more of the transmembrane PEXEL proteins may form the receptor in the PI3P patch for soluble plasmepsin V-cleaved PEXEL proteins ( Figure 2B).

## The way out

One option is that the PI3P patches and their associated proteins are simply packaged into a specific subset of ER-to-Golgi transport vesicles, and are then transported through the secretory pathway by a series of vesicle budding and fusion events ( Figure 3, pathway A). After transport vesicle fusion at the plasma membrane, the plasma membrane then could either bud vesicles outwardly that subsequently fuse with the PVM ( Figure 3), or there might be a transient fusion of parasite plasma membrane and PVM to transmit the proteins perhaps by interaction of the PI3P patch with a receptor in the PVM ( Figure 3). Release of soluble proteins from the PI3P patch might be triggered by different (ion etc.) conditions in the erythrocyte cytosol. Membrane proteins would be transported by vesicular transport from the PVM to Maurer's clefts where their release from the PI3P patches could be triggered by a PI3P-phosphatase.

Alternatively, similar to what has been observed during autophagosomal membrane formation at the ER, the recruitment of specific proteins to the PI3P patch may lead to an invagination of the ER membrane, resulting in vesicles inside the ER containing the PEXEL proteins ( Figure 3, pathway B). These proteins could then be transported through the secretory pathway as double membrane vesicles (DMVs) whose outer layer would ultimately fuse with the parasite plasma membrane ( Figure 3, 3B). DMVs have been detected in electron micrographs of *P. falciparum*, and fusion with the parasite plasma membrane and vesicle release into the PV have been reported ^5^. The released vesicles might subsequently be able to fuse with the PVM ( Figure 3, 4B).

Either of the transport pathways depicted in Figure 3 would satisfy the Brefeldin A sensitivity of (at least some) protein transport into the erythrocyte ^29, 30^. Either would explain how soluble and transmembrane proteins can be targeted into the host cell using the same signal ^3, 4^. Both scenarios could also explain how proteins without a PEXEL signal or proteins without a signal sequence or transmembrane domain could end up in the erythrocyte: these proteins could be packaged into the PEXEL-protein containing vesicles by interaction with these on the cytoplasmic face of the ER membrane ^6, 31^. Both hypotheses could also explain how the PEXEL signal leads to targeting to the erythrocyte even though the signal itself is cleaved already at the parasite ER ^13^.

If the key decision - entry into the conventional secretory pathway or entry into a distinct export pathway that ultimately leads to arrival in the erythrocyte - is already made during PEXEL protein biogenesis at the parasite ER, this would explain why a protein that has been engineered to contain a conventional signal peptide and an N-terminus equivalent to the cleaved PEXEL signal ends up in the PV, not in the erythrocyte ^9^. The construct with the conventional signal peptide is fully translocated into the secretory pathway, and hence separated from the PI3P patch-associated PEXEL proteins ( Figure 2B). It will therefore, like conventional secretory proteins, follow the classical secretory pathway and be secreted into the PV, from which there seems to be no direct access into the erythrocyte.

That this is true is also confirmed by a carefully done set of experiments by Gehde and colleagues (2008) ^32^. The authors aimed to investigate whether protein folding has an effect on PEXEL protein access to the erythrocyte. They generated fusion proteins that contained the signal peptide and PEXEL region from either the transmembrane protein STEVOR or the soluble protein GBP130 fused to dihydrofolate reductase (DHFR), followed by green fluorescent protein (GFP) and expressed these fusion proteins in *P. falciparum*. They found that in the absence of folate analogues (which promote DHFR folding) these constructs were targeted to the erythrocyte. In the presence of folate analogues, the constructs were found in the PV. Strikingly, only newly synthesized proteins could be transported into the erythrocyte, i.e. it was impossible to chase pre-existing fusion proteins from the PV into the erythrocyte after washout of the folate analogue. The authors' interpretation of the data was that proteins must be unfolded in order to be transported across the PVM into the erythrocyte, that the PVM therefore contained a protein-conducting channel with similar requirements for transport as the Sec61 channel in the ER membrane, and that the time window after synthesis during which proteins were transport-competent was limited.

The scenario depicted in Figure 2B suggests a different interpretation of the data. Immediately after targeting of a PEXEL protein to the Sec61 channel in the ER membrane, there is a competition between full translocation of the fusion protein into the ER lumen and binding of the PEXEL region to PI3P on the cytoplasmic leaflet of the ER membrane, which ultimately leads to an abortion of translocation. At this stage the signal peptide and PEXEL regions of the fusion protein are already synthesized, but the ribosome is still associated with the nascent chain and protein synthesis is still going on (between step 1 and step 2 in Figure 2B, not shown). When the DHFR part of the protein emerges from the ribosome, it is initially located in the ER lumen. In the absence of the folate analogue, the DHFR chain remains sufficiently flexible for the PEXEL signal to interact with PI3P, plasmepsin V cleavage occurs, and translocation is aborted; as a result the fusion protein remains in the cytosol associated with the PI3P patch as in Figure 2B. In the presence of a folate analogue, DHFR will fold tightly in the ER lumen during its synthesis and this will interfere with or override the interaction of PEXEL with PI3P in the cytoplasm. As a result the fusion protein will be fully translocated and end up inside the secretory pathway. That folding accelerates translocation into the ER has been shown ^33^. If protein folding in the ER lumen and PI3P-binding at the cytoplasmic leaflet of the ER membrane interfere with each other this might also explain why some of the PEXEL proteins contain large intrinsically unstructured regions ^34^.

On the whole the model depicted in Figure 2B and Figure 3 makes sense of the vast majority of the available data on trafficking of proteins from *P. falciparum* into the host cell and suggests that the decision of where to go is made early during biogenesis of exported proteins at the parasite ER membrane. My hypothesis has a number of easily testable elements that might give the research in this field the appropriate direction for a full understanding of the mechanism of protein export from *P. falciparum* into the erythrocyte.

## References

[1] Maier AG Cooke BM Cowman AF: Malaria parasite proteins that remodel the host erythrocyte. *Nat Rev Microbiol.* 2009;7(5):341–354. 10.1038/nrmicro2110 19369950

[2] Goldberg DE Cowman AF: Moving in and renovating: exporting proteins from *Plasmodium* into host erythrocytes. *Nat Rev Microbiol.* 2010;8(9):617–621. 10.1038/nrmicro2420 20706280

[3] Marti M Good RT Rug M: Targeting malaria virulence and remodeling proteins to the host erythrocyte. *Science.* 2004;306(5703):1930–1933. 10.1126/science.1102452 15591202

[4] Hiller NL Bhattacharjee S van Ooij C: A host-targeting signal in virulence proteins reveals a secretome in malarial infection. *Science.* 2004;306(5703):1934–1937. 10.1126/science.1102737 15591203

[5] Cooke BM Lingelbach K Bannister LH: Protein trafficking in *Plasmodium falciparum*-infected red blood cells. *Trends Parasitol.* 2004;20(12):581–589. 10.1016/j.pt.2004.09.008 15522668

[6] Römisch K: Protein targeting from malaria parasites to host erythrocytes. *Traffic.* 2005;6(8):706–709. 10.1111/j.1600-0854.2005.00310.x 15998325

[7] Lingelbach K Przyborski JM: The long and winding road: Protein trafficking mechanisms in the *Plasmodium falciparum* infected erythrocyte. *Mol Biochem Parasitol.* 2006;147(1):1–8. 10.1016/j.molbiopara.2006.01.014 16540187

[8] Boddey JA Moritz RL Simpson RJ: Role of the *Plasmodium* export element in trafficking parasite proteins to the infected erythrocyte. *Traffic.* 2009;10(3):285–299. 10.1111/j.1600-0854.2008.00864.x 19055692 PMC2682620

[9] Boddey JA Hodder AN Günther S: An aspartyl protease directs malaria effector proteins to the host cell. *Nature.* 2010;463(7281):627–631. 10.1038/nature08728 20130643 PMC2818761

[10] Russo I Babbit S Muralidharan V: Plasmepsin V licenses *Plasmodium* proteins for export into the host erythrocyte. *Nature.* 2010;463(7281):632–636. 10.1038/nature08726 20130644 PMC2826791

[11] Chang HH Falick AM Carlton PM: N-Terminal processing of proteins exported by malaria parasites. *Mol Biochem Parasitol.* 2008;160(2):107–115. 10.1016/j.molbiopara.2008.04.011 18534695 PMC2922945

[12] Klemba M Goldberg DE: Characterization of plasmepsin V, a membrane-bound aspartic protease homolog in the endoplasmic reticulum of *Plasmodium falciparum.* *Mol Biochem Parasitol.* 2005;143(2):183–191. 10.1016/j.molbiopara.2005.05.015 16024107

[13] Bhattacharjee S Stahelin RV Speicher KD: Endoplasmic reticulum PI(3)P lipid binding targets malaria proteins to the host cell. *Cell.* 2012;148(1–2):201–212. 10.1016/j.cell.2011.10.051 22265412 PMC3268671

[14] De Koning-Ward TF Gilson PR Boddey JA: A newly discovered protein export machine in malaria parasites. *Nature.* 2009;459(7249):945–949. 10.1038/nature08104 19536257 PMC2725363

[15] Bullen HE Charnaud SC Kalanon M: Biosynthesis, localization, and macromolecular arrangement of the *Plasmodium falciparum* translocon of exported proteins (PTEX). *J Biol Chem.* 2012;287(11):7871–7894. 10.1074/jbc.M111.328591 22253438 PMC3318755

[16] Arnesen T: Towards a functional understanding of protein N-Terminal Acetylation. *PLoS Biol.* 2011;9(5):e1001074. 10.1371/journal.pbio.1001074 21655309 PMC3104970

[17] Forte GM Pool MR Stirling CJ: N-Terminal acetylation inhibits protein targeting to the endoplasmic reticulum. *PLoS Biol.* 2011;9(5):e1001073. 10.1371/journal.pbio.1001073 21655302 PMC3104963

[18] Fujiki Y Hubbard AL Fowler S: Isolation of intracellular membranes by means of sodium carbonate treatment: application to endoplasmic reticulum. *J Cell Biol.* 1982;93(1):97–102. 10.1083/jcb.93.1.97 7068762 PMC2112113

[19] Gillooly DJ Morrow IC Lindsay M: Localization of phosphatidylinositol 3-phosphate in yeast and mammalian cells. *EMBO J.* 2000;19(17):4577–4588. 10.1093/emboj/19.17.4577 10970851 PMC302054

[20] Lindmo K Stenmark H: Regulation of membrane traffic by phosphoinositide 3-kinases. *J Cell Sci.* 2006;119(Pt 4):605–614. 10.1242/jcs.02855 16467569

[21] Axe EL Walker SA Manigava M: Autophagosome formation from membrane compartments enriched in phosphatidylinositol 3-phosphate and dynamically connected to the endoplasmic reticulum. *J Cell Biol.* 2008;182(4):685–701. 10.1083/jcb.200803137 18725538 PMC2518708

[22] Matsunaga K Morita E Saitoh T: Autophagy requires endoplasmic reticulum targeting of the PI3-kinase complex via Atg14L. *J Cell Biol.* 2010;190(4):511–521. 10.1083/jcb.200911141 20713597 PMC2928018

[23] Dautin N Bernstein HD: Protein secretion in gram-negative bacteria via the autotransporter pathway. *Annu Rev Microbiol.* 2007;61:89–112. 10.1146/annurev.micro.61.080706.093233 17506669

[24] Papakrivos J Newbold CI Lingelbach K: A potential novel mechanism for the insertion of a membrane protein revealed by a biochemical analysis of the *Plasmodium falciparum* cytoadherence molecule PfEMP-1. *Mol Microbiol.* 2005;55(4):1272–1284. 10.1111/j.1365-2958.2004.04468.x 15686570

[25] Przyborski JM Miller SK Pfahler JM: Trafficking of STEVOR to the Maurer’s clefts in *Plasmodium falciparum*-infected erythrocytes. *EMBO J.* 2005;24(13):2306–17. 10.1038/sj.emboj.7600720 15961998 PMC1173160

[26] Simonsen A Stenmark H: Self-eating from an ER-associated cup. *J Cell Biol.* 2008;182(4):621–622. 10.1083/jcb.200807061 18725534 PMC2518700

[27] Gaur D Chitnis CE: Molecular interactions and signaling mechanisms during erythrocyte invasion by malaria parasites. *Curr Opinion Microbiol.* 2011;14(4):422–428. 10.1016/j.mib.2011.07.018 21803641

[28] Goder V Spiess M: Molecular mechanism of signal sequence orientation in the endoplasmic reticulum. *EMBO J.* 2003;22(14):3645–3653. 10.1093/emboj/cdg361 12853479 PMC165631

[29] Benting J Mattei D Lingelbach K: Brefeldin A inhibits transport of the glycophorin-binding protein from *Plasmodium falciparum* into the host erythrocyte. *Biochem J.* 1994;300(Pt 3):821–826. 8010965 10.1042/bj3000821 PMC1138239

[30] Wickham ME Rug M Ralph SA: Trafficking and assembly of the cytoadherence complex in *Plasmodium falciparum*-infected human erythrocytes. *EMBO J.* 2001;20(20):5636–5649. 10.1093/emboj/20.20.5636 11598007 PMC125667

[31] Spielmann T Gilberger TW: Protein export in malaria parasites: do multiple export motifs add up to multiple export pathways? *Trends Parasitol.* 2010;26(1):6–10. 10.1016/j.pt.2009.10.001 19879191

[32] Gehde N Hinrichs C Montilla I: Protein unfolding is an essential requirement for transport across the parasitophorous vacuolar membrane of *Plasmodium falciparum*. *Mol Microbiol.* 2009;71(3):613–28. 10.1111/j.1365-2958.2008.06552.x 19040635

[33] Scheper W Thaminy S Kais S: Coordination of *N*-glycosylation and protein translocation across the Endoplasmic Reticulum membrane by Sss1 protein. *J Biol Chem.* 2003;278(39):37998–38003. 10.1074/jbc.M300176200 12860997

[34] Feng ZP Zhang X Han P: Abundance of intrinsically unstructured proteins in *P. falciparum* and other apicomplexan parasite proteomes. *Mol Biochem Parasitol.* 2006;150(2):256–267. 10.1016/j.molbiopara.2006.08.011 17010454

