# Supplementary material for: Diversion at the ER: How Plasmodium falciparum exports proteins into host erythrocytes
Source: F1000Res. 2012 Dec 7;1:12. Originally published 2012 Aug 22. [Version 2] doi: 10.12688/f1000research.1-12.v2 (PMC4382842; doi:10.12688/f1000research.1-12.v2)
Supplement: Peer review history

# Peer review: Referee response for version 1

**DOI:** 10.5256/f1000research.108.r345

**Published:** 2012-10-30

**Authors:** Tobias Spielman

**Affiliations:** 1 Bernhard Nocht Institute for Tropical Medicine, Hamburg, Germany

This is a very thought provoking review on protein export in the malaria parasite *Plasmodium falciparum*. The proposed model is centered on the fact that in other systems, N-terminal acetylation of proteins is found in the cytosol, not the ER lumen.

If this is adopted for *P. falciparum*, it literally turns the current model for protein export inside out: instead of the previously assumed binding of the PEXEL export motif to PI3P and subsequent processing of the PEXEL within the ER, cytosolic acetylation of the processed N-terminus would place these events to the outside of the ER. As these events are considered to be the key requirements for export, this is of great importance to understand protein export in malaria parasites.

The review then draws on previously published models for vesicular trafficking in *P. falciparum* and autophagy-related processes to propose pathways for the de-PEXELed protein on the outer ER leaflet to reach the host cell. For instance this would make possible a previously considered elegant model (REF 7) of serial events of vesicular budding and fusion leading to delivery of the protein into the host cell without direct crossing of any membranes.

Overall this is a well written review with a clear hypothesis that adds an important new angle for the field to consider. Although the proposed model fits a lot of the data on protein export, there are also some discrepancies that become obvious when delving into the details. There are a number of points I would like to raise for discussion:

- The explanation why the mDHFR fusion constructs (REF 32) do not challenge the hypothesis (page 5, third and fourth paragraph) is difficult to understand. Is it assumed that the mDHFR domain is in the ER lumen while the PEXEL binds to the outside of the ER and that this binding would be able to rescue the unfolded domain but not the folded version out of the ER? In this case it is not entirely clear how this fits into the model for translocation of proteins into the ER. A graphical explanation of the topology of this situation with the mDHFR fusion protein and how it can ensue would be helpful. This is important, as this part of the review is central to discounting the prominently proposed protein translocation at the PVM (REF 14). As an aside on this topic: while it is true that functional evidence for the PVM translocon is lacking, it may be worth noting that there is some earlier evidence for ATP-dependent translocation of an exported protein across the PVM (Ansorge et al., 2006; Biochem J).

- Boddey et al. (REF 8) carried out a detailed analysis of 2 proteins with mutated PEXELs. For one of these proteins they found a signal peptidase-cleaved species that was retained in/at the ER. Despite an R->A mutation in the PEXEL (position 1) this protein was N-acetylated. Thus, in this case acetylation seemed independent of PEXEL mediated PI3P binding and PMV cleavage. As the signal peptide is usually cleaved during translocation, this could also indicate N-acetylation after ER entry. For the second protein, the PEXEL cleaved E->A mutation (position 5) was analysed. This protein was found in the PV despite being N-acetylated. This would even more strongly argue that protein entering the ER can still be acetylated. Finally, N-acetylation also occurred with the signal peptidase-cleaved mutated PfEMP3 trafficked to the PV (REF 9), a construct mentioned in this review as an example not entering the proposed pathway for export. These findings seem to be at odds with the proposed model.

- The data on the exact localisation of plasmepsin V, while not exactly excluding the possibility suggested in this review (page 2), could as well indicate a localisation within the ER. If I interpret the images from the episomally expressed constructs from REF 10 correctly, then the signal peptide mediates (and is sufficient for) PV targeting and thus also for ER entry. This would indicate a failure of ER retention rather than lack of ER targeting in the mutants lacking the transmembrane domain. The conclusion here that this data ‘demonstrates that it is not a signal sequence’ may therefore not be correct. It is also not clear why PMV would possess an N-terminal hydrophobic region at all if it is targeted by its C-terminal hydrophobic region (P. falciparum contains components for the insertion of tailanchored proteins which do not require a signal peptide).

- The explanation for PEXEL negative protein (PNEP) export is a bit weak (page 5, first paragraph). Binding of PNEPs to PEXEL proteins as a means for export could occur in most models for export and is no particular feature of the alternative proposed here. In fact, for this model, I would expect PNEPs not to require a hydrophobic region at all.

- As a general consideration. If N-acetylation in Plasmodium parasites is expected to strictly follow the current cell biological concepts from other systems, then this should equally be considered for the vesicle trafficking needed as a result of this (i.e. to make possible the export of a protein on the outer face of the ER). In particular this concerns how proteins get across the PVM. There is no tangible evidence for the presence of the components known to mediate vesicular trafficking (e.g. coats, SNAREs, rabs etc) beyond the parasite plasma membrane (absence of evidence of course is not necessarily evidence for absence, but in contrast many classical components in anterograde and retrograde vesicular trafficking seem to be present within the parasite, see e.g. Deponte et al., MBP 2012). How the vesicle trafficking necessary to suit the model is achieved may therefore be a similarly (or even more) enigmatic cell biological problem than how proteins could be acetylated within the ER.

- Although various types of vesicles have been documented in the host cell (on the morphological level, directed trafficking has not been demonstrated), I am unaware of any report convincingly showing vesicles in the PV. The close proximity of the PPM and PVM seem to make vesicle trafficking not the most suitable way for this. Is there any precedent for vesicular trafficking in such a situation?

- Although an opinion article, alternatives to cytoplasmic acetylation could perhaps be discussed. Possibly there still is an unknown N-terminal acetylation activity in the ER of Plasmodium parasites (for instance there is one GNAT domain containing protein, PF14_0350, with 2 hydrophobic segments). Or, at least as a formal possibility, can it be entirely excluded that the observed N-acetylation of processed PEXEL proteins is non-physiological and occurs during affinity purification of these proteins from parasite extracts? This would require posttranslational N-terminal acetylation activities which, at least in yeast, seem to exist (Helsens et al., 2011 J Proteome Res).

I have read this submission. I believe that I have an appropriate level of expertise to confirm that it is of an acceptable scientific standard.

# Response

**Published:** 2012-11-20

**Authors:** Karin Romisch

**Affiliations:** Department of Microbiology, Faculty of Medicine, Saarland University, Germany

Dear Dr. Spielmann,thank you very much for your careful review of my opinion article. I will answer your questions in the order you raised them:

You understood correctly what I was saying about the methotrexate-mediated DHFR folding in the ER lumen competing with PEXEL binding to PI3P and plasmepsin V on the cytoplasmic face of the ER membrane. I had considered adding a drawing to the paper, but in the end I decided against it, because I felt it would distract the readers from the central issues I discuss. The paper you mention ( Ansorge *et al.*, Biochem J 1996) describes elegant work from the Lingelbach lab in which they showed ATP-dependent release of GBP from streptolysin O-permeabilized infected erythrocytes. During the permeabilization, the vacuolar membrane stays intact and GBP remains protease-protected. Upon addition of ATP and cytosol GBP is released from the PV and becomes protease-accessible. This could either be ATP-dependent translocation of GBP across the PVM, or it could be ATP-induced fusion of GBP-containing vesicles with the PVM; the data is compatible with either model.

The N-acetylation of signal-peptide cleaved proteins is indeed puzzling, and I do not have a physiological explanation for it. As you point out, one possibility is that this is a post-lysis artefact and therefore not relevant for the topology of the targeting of proteins exported from *P. falciparum* to the host cell. Topology predictions for the transmembrane GNAT-domain protein you mention are contradictory using several programmes, so it is unclear which side of the membrane the active site of the protein would be. N-acetylation within the secretory pathway, however, would also require import of Acetyl-CoA across a membrane (ER, Golgi) and a transporter. Is there such a protein? – It also remains unclear whether N-acetylation is actually part of the export signal for erythrocyte targeted proteins. Given that there is no conservation of the residue which is modified in the cleaved PEXEL (the x before the E/Q), it seems that this modification may not be important which is why I prefer not to expand this part of my review.

Thank you very much for pointing out my failure to understand the data characterizing the function of the hydrophobic N-terminal domain of plasmepsin V in Russo *et al.*, Nature 2010. To me the images of the YFP staining and the SP-YFP staining looked fairly similar and I took the text in the paper “the transmembrane domain, but no other portions of plasmepsin V were sufficient to target a reporter to the ER” to mean YFP and SP-YFP were both cytosolic and remained inside the parasite. But I agree if you blow up the images, the hydrophobic region at the N-terminus fused to YFP results in ring-shaped staining around the cytoplasm which could be the PV, or the parasite plasma membrane. I will modify this part of the text in the next version of my review. This does not, however, alter my prediction that the active site of plasmepsin V will be in the cytoplasm. The N-terminal hydrophobic domain does not have a signal peptide cleavage site, so it is likely a transmembrane domain, and both TMHMM and TopPred predict it to insert with the N-terminus in the ER lumen such that the subsequent soluble region containing the active site would be in the cytoplasm. But this is all *in silico*, and since it is a really important issue, I sincerely hope that somebody will characterize the actual topology of the enzyme in the ER membrane biochemically.

Regarding vesicles in the PV and vesicle fusion in the absence of SNAREs etc.: If these vesicles were present in the PV only transiently and given the very narrow lumen of the PV it would be exceedingly difficult to capture images of such vesicles by electron microscopy unless their fusion were inhibited by some means. Perhaps they could be accumulated in SLO-permeabilized infected erythrocytes as in Ansorge et al., 1996. There are examples of vesicle fusion in the absence of the conventional eukaryotic fusion machinery: gram-negative bacteria can shed outer membrane derived vesicles which are subsequently either taken up by or fuse with eukaryotic cells. In many, but not all, cases these vesicles contain pathogenicity factors. It is conceivable that transport vesicles containing host cell-targeted *P. falciparum* proteins fuse with the PVM (which is derived from the erythrocyte plasma membrane) in a manner similar to these bacterial vesicles. The ATP-dependent vesicle fusion observed by Ansorge et al, 1996, may actually be mediated by the ‘translocation complex’ isolated by Cowman’s lab which contains a protein with homology to a bacterial pore-forming protein.

One final argument against the lumen of the ER being involved in the biogenesis of proteins exported into the erythrocyte is the apparent absence of essential elements of the Unfolded Protein Response (UPR) in *P. falciparum*. The UPR is switched on in all eukaryotes under conditions that require an expansion of protein secretory capacity (e.g. insulin secretion in the pancreas, or maturation of plasma cells for antibody production) and is essential under those circumstances. One would expect *P. falciparum* to have to expand its secretory capacity when upon invasion of an erythrocyte it suddenly has to secrete hundreds of proteins into the host cell. In yeast, the UPR is mediated by a single transmembrane protein in the ER, Ire1, which upon accumulation of misfolded proteins oligomerizes and splices the mRNA for a transcription factor, Hac1, which then activates transcription of ER chaperones etc. This branch of the UPR is highly conserved in eukaryotes. Mammals have two further UPR sensors in the ER, PERK and ATF6. The last time I looked (ca 2006) in the *P. falciparum* genome there were no ORFs with significant similarity to the central UPR transcription factor Hac1 or its mammalian orthologue XBP-1, or ATF6, and only proteins with weak similarity to Ire1 and PERK. So either *P. falciparum *has evolved a novel way to adapt to increase of the load on the secretory pathway, or it does not experience much variation in the flux through the ER, because it does not use the conventional secretory pathway for secretion of host cell targeted proteins.

# Peer review: Referee response for version 1

**DOI:** 10.5256/f1000research.108.r344

**Published:** 2012-08-28

**Authors:** Norma Andrews

**Affiliations:** 1 Department of Cell Biology and Molecular Genetics, University of Maryland, MD, 20742, USA

This is a very interesting and compelling article. In a simple and extremely clear style Karin Romisch does a beautiful job placing protein export by intracellular malaria parasites into a solid cell biological context.

She corrects important topological misconceptions (unfortunately common in the parasitology/infectious disease fields), acknowledges other authors with similar ideas, and overall makes a crystal clear and important contribution to our understanding of one of the most fascinating problems in protein transport to emerge in recent years.

I have read this submission. I believe that I have an appropriate level of expertise to confirm that it is of an acceptable scientific standard.

# Peer review: Referee response for version 1

**DOI:** 10.5256/f1000research.108.r343

**Published:** 2012-08-28

**Authors:** Malcolm McConville

**Affiliations:** 1 Department of Biochemistry and Molecular Biology, The University of Melbourne, Melbourne, Australia

The author proposes a new model for how proteins in intraerythrocytic stages of the malarial parasite could be transported from the parasite endoplasmic reticulum to the host cell cytoplasm.

The new model is compatible with much of the existing Plasmodium protein export literature and incorporates additional mechanistic insights from other protein trafficking process. In particular, a compelling case is made for key processing and transport steps in Plasmodium protein export occurring on the cytoplasmic leaflet of the ER and transport vesicles based on the known topology of phosphatidylinositol-3-phosphate and protein N-acetylation reactions, both of which have been shown to be involved in plasmodium protein export. Another major feature of this model is that exported soluble and membrane proteins are transported from the parasite plasma membrane to the parasitophorous vacuole via membrane vesicles. The internalization of proteins into transport vesicles draws on precedents observed during autophagy and is plausible, but raises important questions about the role of a parasite-derived multi-protein complex in the PV membrane that is thought to translocate parasite proteins across this membrane.

I have read this submission. I believe that I have an appropriate level of expertise to confirm that it is of an acceptable scientific standard.
